# An abdominal obesity missense variant in the adipocyte thermogenesis gene *TBX15* is implicated in adaptation to cold in Finns

**DOI:** 10.1016/j.ajhg.2024.10.001

**Published:** 2024-11-07

**Authors:** Milena Deal, Asha Kar, Seung Hyuk T. Lee, Marcus Alvarez, Sandhya Rajkumar, Uma Thanigai Arasu, Dorota Kaminska, Ville Männistö, Sini Heinonen, Birgitta W. van der Kolk, Ulla Säiläkivi, Tuure Saarinen, Anne Juuti, Jussi Pihlajamäki, Minna U. Kaikkonen, Markku Laakso, Kirsi H. Pietiläinen, Päivi Pajukanta

**Affiliations:** 1 Department of Human Genetics, University of California, Los Angeles, Los Angeles, CA, USA; 2 Bioinformatics Interdepartmental Program, UCLA, Los Angeles, CA, USA; 3 A. I. Virtanen Institute for Molecular Sciences, University of Eastern Finland, Kuopio, Finland; 4 Institute of Public Health and Clinical Nutrition, University of Eastern Finland, Kuopio, Finland; 5 Department of Medicine, Division of Cardiology, David Geffen School of Medicine at UCLA, Los Angeles, CA, USA; 6 Department of Medicine, University of Eastern Finland and Kuopio University Hospital, Kuopio, Finland; 7 Obesity Research Unit, Research Program for Clinical and Molecular Metabolism, Faculty of Medicine, University of Helsinki, Helsinki, Finland; 8 Department of Abdominal Surgery, Abdominal Center, Helsinki University Hospital and University of Helsinki, Helsinki, Finland; 9 Department of Medicine, Endocrinology and Clinical Nutrition, Kuopio University Hospital, Kuopio, Finland; 10 HealthyWeightHub, Endocrinology, Abdominal Center, Helsinki University Central Hospital and University of Helsinki, Helsinki, Finland; 11 Institute for Precision Health, David Geffen School of Medicine at UCLA, Los Angeles, CA, USA

**Keywords:** single nucleus RNA sequencing, abdominal obesity, subcutaneous adipose tissue, SAT, T-box transcription factor 15, TBX15, trans regulation, waist-hip ratio adjusted for body mass index, WHRadjBMI, selection, thermogenesis, population genetics, adipocyte hypertrophy

## Abstract

Mechanisms of abdominal obesity GWAS variants have remained largely unknown. To elucidate these mechanisms, we leveraged subcutaneous adipose tissue (SAT) single nucleus RNA-sequencing and genomics data. After discovering that heritability of abdominal obesity is enriched in adipocytes, we focused on a SAT unique adipocyte marker gene, the transcription factor *TBX15*, and its abdominal obesity-associated deleterious missense variant, rs10494217. The allele frequency of rs10494217 revealed a north-to-south decreasing gradient, with consistent significant F_ST_ values observed for 25 different populations when compared to Finns, a population with a history of genetic isolation. Given the role of Tbx15 in mouse thermogenesis, the frequency may have increased as an adaptation to cold in Finns. Our selection analysis provided significant evidence of selection for the abdominal obesity risk allele T of rs10494217 in Finns, with a north-to-south decreasing trend in other populations, and demonstrated that latitude significantly predicts the allele frequency. We also discovered that the risk allele status significantly affects SAT adipocyte expression of multiple adipocyte marker genes in *trans* in two cohorts. Two of these *trans* genes have been connected to thermogenesis, supporting the thermogenic effect of the *TBX15* missense variant as a possible cause of its selection. Adipose expression of one *trans* gene, a lncRNA, *AC002066.1*, was strongly associated with adipocyte size, implicating it in metabolically unhealthy adipocyte hypertrophy. In summary, the abdominal obesity variant rs10494217 was selected in Finns, and individuals with the risk allele have *trans* effects on adipocyte expression of genes relating to thermogenesis and adipocyte hypertrophy.

## Teaser

The transcription factor *Tbx15* has been implicated in mouse thermogenesis. Here, the authors identified signals of selection in Finns for a *TBX15* missense variant associated with abdominal obesity and discovered that the obesity risk allele of this variant affects adipocyte expression of 13 genes in *trans*, including known thermogenesis genes.

## Introduction

The vast majority of adults in the United States and many other countries globally are overweight or obese, defined as a body mass index (BMI) of ≥25.^1^ Waist-hip ratio adjusted for BMI (WHRadjBMI) is a well-established proxy of abdominal obesity,^2^^,^^3^ a form of central obesity closely associated with obesity-induced cardiometabolic comorbidities,^4^ including type 2 diabetes (T2D).^5^ While abdominal obesity is known to be a sexually dimorphic trait,^6^^,^^7^ there are knowledge gaps in the cell-type mechanisms contributing to these differences between the sexes.

Abdominal obesity is associated with dysfunctional adipose tissue.^8^ Specifically, the excessive accumulation of fat in obesity can be characterized by adipocyte hypertrophy, or expansion of fat cells,^9^ closely linked to adverse cardiometabolic conditions.^9^^,^^10^ Although metabolically healthier expansion of adipose tissue may take place through adipocyte hyperplasia, i.e., generation of new small adipocytes, rather than hypertrophy,^9^ the cell-type transcriptional mechanisms by which adipocytes favor one path over the other are still elusive.

Here we used subcutaneous adipose tissue (SAT) single nucleus RNA-sequencing (snRNA-seq) data from two independent Finnish cohorts to identify cell-type genetic and molecular mechanisms underlying abdominal obesity. We discovered that adipocytes are the only SAT cell type with enriched heritability for abdominal obesity, further refining a previous SAT tissue-level study.^6^ By leveraging previous large-scale WHRadjBMI genome-wide association study (GWAS) data,^11^ we then searched for abdominal obesity GWAS variants that reside within the coding regions of the SAT unique adipocyte marker genes to enable the assessment of their effects on expression at the cell-type resolution based on the abdominal obesity GWAS risk allele status. This design focused us on an abdominal obesity GWAS missense variant, rs10494217 (GenBank: NM_001330677.2) (c.466C>A [p.His156Asn]), predicted to be deleterious and residing in *TBX15*, a key adipose transcription factor (TF)^8^ that has been implicated for white adipocyte browning and thermogenesis in previous mouse studies.^12^^,^^13^ Specifically, inguinal adipocyte browning was significantly impaired in a mouse model with a conditional *Tbx15* knockout, and the mice experienced additional weight gain in response to high-fat diets.^12^ Knockdown of *Tbx15* mRNA impaired differentiation in brown adipocytes and inguinal white adipocytes.^13^ In addition, our previous paper in humans has identified a SAT co-expression network correlated with abdominal obesity and regulated by TBX15 using bulk RNA-seq analysis followed by siRNA knockdown experiments.^14^ We also determined that *TBX15* is the likely susceptibility gene at the *TBX15/WARS2* locus.^14^ Finally, *TBX15* is also known to display sex-specific effects^14^ and may thus influence the sexual dimorphisms of abdominal obesity.^6^^,^^7^

One hypothesis for the genetic origin of obesity is that it is partly due to alleles that originally grew to frequency in a different environment, such as one with repeated famine and starvation, but present-day these alleles pose a health risk in the current environment with plentiful availability of high-fat and high-sugar food. This “thrifty variant” hypothesis was proposed in the case of selection of *CREBRF* in Samoans.^15^ Thermogenesis dissipates energy as heat, burning fat and leading to a rise in core body temperature, and here we hypothesized that the rs10494217 abdominal obesity risk allele T of *TBX15* increases body fat to provide more insulation and fuel to thermogenesis and cold resistance. Accordingly, the T allele of rs10494217 could have acted as thermogenically beneficial even though it is metabolically harmful. Thus, it may have been selected for in the populations where increasing thermogenesis could previously have increased fitness while obesity was likely rare due to the sparsity of food and common famines caused by crop failures in northern latitudes.

We investigated this hypothesis and first observed that there is a north-to-south gradient and a large variation in the allele frequencies of the identified abdominal obesity GWAS missense variant in *TBX15* across global populations from different ancestries when compared to Finns, a well-known genetic isolate with a distinct population history that includes multiple bottlenecks and displays founder effects in rare monogenic diseases.^16^^,^^17^ This observation made us search for a signal of selection in the Finns versus the tens of population groups available in the UK Biobank (UKB).^18^ We also assessed downstream effects of the variant on expression of unique adipocyte marker genes in the two SAT snRNA-seq cohorts based on the risk allele status, with the goal to identify unique adipocyte marker genes affected by these *trans* effects of the risk allele. Ultimately, we identified a significant signal of selection acting on the abdominal obesity GWAS missense variant in *TBX15* that also affects gene expression of multiple SAT unique adipocyte marker genes in *trans*, including expression of genes linked to adipocyte hypertrophy, a metabolically unhealthy consequence of obesity.

## Methods

### Study cohorts

#### Kuopio Obesity Study

The existing Kuopio Obesity Study (KOBS) cohort comprises Finnish individuals with obesity who underwent bariatric surgery.^19^^,^^20^ Participants were originally recruited at the University of Eastern Finland and Kuopio University Hospital, Kuopio, Finland. The KOBS participants have either a pre-surgery BMI of ≥40 kg/m^2^ or 35 kg/m^2^ with a significant obesity comorbidity, such as type 2 diabetes (T2D).^19^^,^^20^ In this study, we used subcutaneous adipose tissue (SAT) single nucleus RNA-sequencing (snRNA-seq) data from 8 KOBS participants with obesity, available^21^ in the NIH Gene Expression Omnibus (GEO), under accession number GSE269926. These individuals were genotyped using the Infinium Global Screening Array-24 v1 (Illumina), and their clinical characteristics were measured as described previously.^22^ The study was approved by the local ethics committee, and all participants gave written informed consent. All research conformed to the principles of the Declaration of Helsinki.

#### RYSA cohort

The RYSA bariatric surgery cohort consists of Finnish individuals with obesity (*n* = 68). Individuals were recruited at the Helsinki University Hospital, Helsinki, Finland, as described previously.^23^ In this study, we used SAT snRNA-seq data collected during bariatric surgery from 68 individuals from the RYSA cohort. These individuals were also genotyped using the Infinium Global Screening Array-24 v1 (Illumina). The study was approved by the local ethics committee, and all participants gave written informed consent. All research conformed to the principles of the Declaration of Helsinki.

#### CRYO twin cohort and Finnish BMI-discordant twin cohort

We used existing SAT snRNA-seq data (*n* = 15) from the Finnish CRYO Twin cohort,^14^^,^^24^ available in GEO, under accession number GSE236708. We also used previously generated SAT bulk RNA-seq data^25^ and adipocyte size measurements^26^ from the Finnish BMI-discordant MZ twin cohort (total *n* = 88 with both SAT bulk RNA-seq data and adipocyte size measurements available for study). This BMI-discordant MZ twin cohort was recruited from the population-based Finnish twin cohort (FTC)^27^^,^^28^^,^^29^ by having a large intrapair difference in BMI (ΔBMI ≥ 3 kg/m^2^). Monozygosity was confirmed by testing genome-wide SNP array data, as described previously.^30^ The CRYO Twin and BMI-discordant MZ twin participants were recruited at the University Hospital of Helsinki, Helsinki, Finland. The studies were approved by the local ethics committee, and all participants gave written informed consent. All research conformed to the principles of the Declaration of Helsinki.

#### Metabolic syndrome in men cohort

The METSIM (metabolic syndrome in men) cohort consists of Finnish men (*n* = 10,197) whose DNAs were genotyped using OmniExpress (Illumina) genotyping arrays.^31^ Individuals were recruited at the University of Eastern Finland and Kuopio University Hospital, Kuopio, Finland. In this study, we used previously generated genotype data from the Finnish METSIM cohort (*n* = 6,738 men with 3^rd^ degree unrelatedness defined by KING v.2.3.2^32^ with --unrelated --degree 3).^14^ The study was approved by the local ethics committee and all participants gave written informed consent. All research conformed to the principles of the Declaration of Helsinki.

#### UK Biobank

We used imputed genotype data from the UK Biobank (UKB), which comprises 502,617 participants.^33^ Genotyping was performed using either Affymetrix or Applied Biosystems UK Biobank Axiom genotyping technology.^33^ Imputation was performed using the Haplotype Reference Consortium and the merged UK10K and 1000 Genomes phase 3 reference panel.^18^^,^^33^ We restricted our analyses to 483,560 individuals who were unrelated to account for potential confounding from relatedness. The data that support the findings in this manuscript are available from the UK Biobank. However, restrictions apply to the availability of these data, which were used in this study under UK Biobank Application number 33934. UK Biobank data are available for bona fide researchers through the application process (https://www.ukbiobank.ac.uk/learn-more-about-uk-biobank/contact-us). All UKB participants provided written informed consent.^33^

### Genotype quality control and imputation in KOBS, RYSA, and METSIM

We used genotype data from the 8 KOBS participants with SAT snRNA-seq data to demultiplex the snRNA-seq data (see below). Prior to imputation, we performed quality control (QC) on the KOBS genotype data using PLINK v.1.9.^34^ We removed SNPs that were monomorphic, unmapped, strand ambiguous, were not in Hardy-Weinberg equilibrium (HWE) with *p* < 10^−6^, or had missingness >2%.^14^ We used the --sex-check function in PLINK^34^ to validate with the reported sex of each individual. We imputed the genotype data using the Michigan Imputation Server using the Haplotype Reference Consortium (HRC) version r1.1 2016 as a reference panel after removing variants with allele mismatch to the reference and duplicate variants.^14^ We performed haplotype phasing using Eagle v.2.4^35^ and imputation using minimac4^36^ after doing strand flips or allele switches to match the reference. We then removed SNPs from the imputed genotype data with HWE *p* < 10^−6^ and imputation score R^2^ < 0.3.

For the RYSA replication cohort (*n* = 68), the genotype data for rs10494217 was generated as part of the Infinium Global Screening Array-24 v1 (Illumina), also used in KOBS, performing the QC, imputation, and QC of the imputed genotyped data as described above.

We used existing genotype data generated using an Illumina HumanOmniExpress BeadChip from 6,738 unrelated METSIM participants in this study^14^^,^^31^ and performed QC as described above. We uploaded the high-quality genotypes to the TOPMed imputation server^36^ where duplicate variants and variants with allele mismatch with the reference panel were removed. In addition, strand flips or allele switches needed to match the reference panel were performed by the server. Haplotype phasing was done using Eagle v.2.4^35^ and genotype imputation was performed against the TOPMed reference panel version r2^37^ using minimac4.^36^ We performed QC on the imputed genotype data as described above.

### Nuclei isolation and snRNA-seq of SAT in KOBS and RYSA

We performed snRNA-seq on the SAT biopsies collected during bariatric surgery from 8 individuals of the KOBS cohort, as described previously.^14^^,^^21^ First, we pooled together the 8 biopsy samples, each with approximately 100 mg of tissue, then isolated the nuclei after pooling.^14^ After staining with trypan blue and DAPI, we measured the nuclei quality and concentration using Countess II FL Automated Cell Counter. For the library construction, we used the Single Cell 3′ Reagent Kit v.3.1 (10× Genomics). We used the Agilent Bioanalyzer to analyze the quality of the cDNA and gene expression library. We sequenced the library using an Illumina NovaSeq SP with 600 million read pairs as the target sequencing depth.

We also performed snRNA-seq of the SAT samples collected during bariatric surgery from the 68 RYSA participants. These data were generated as part of the full RYSA cohort, where samples of 8 were pooled together for each batch for the nuclei isolation. We isolated nuclei and generated libraries as described above for the snRNA-seq. We sequenced all batches together using an Illumina NovaSeq S4 and had 400 million reads per batch as the targeted sequencing depth.

### Processing of SAT snRNA-seq data in KOBS

We processed the SAT snRNA-seq data from the 8 individuals of the KOBS cohort as previously described with minor modifications.^14^ Briefly, using STARSolo of STAR v.2.7.5a,^38^ we aligned the sequence reads to the GRCh37 human genome reference using the GENCODE v.19 annotation.^39^ To account for full pre-mRNA transcripts, the STAR --soloFeatures GeneFull option was used. We used DIEM v.2.4.0^40^ with the default parameters to remove droplets containing background RNA, then filtered droplets further using Seurat v.4.3.0.^41^ To demultiplex droplets from the pooled 8 samples, we employed demuxlet from popscle software tool^42^ using genotype data and --min-MQ 30 to find the individual of origin of each droplet using maximum likelihood. We excluded doublets and ambiguously assigned droplets. We log-normalized the count data using the default scaling factor of 10,000, then used FindVariableGenes to identify the top 2,000 variable genes. Then the normalized read counts were scaled to a mean of 0 and variance 1 and the first 30 principal components (PCs) were calculated for clustering with standard Louvain clustering and a clustering resolution of 0.8. Finally, we used SingleR v.1.2.4^43^ to annotate each droplet with a cell type, using the same reference datasets as previously.^14^

### Processing of SAT snRNA-seq data in RYSA

We aligned the FASTQ files of the raw snRNA-seq data for each batch using the GRCh38 reference genome and GENCODE v.42 annotations^44^ using STAR v.2.7.10b.^38^ We accounted for full pre-mRNA transcripts by using the --soloFeatures GeneFull option. The FastQC tool (https://www.bioinformatics.babraham.ac.uk/projects/fastqc/) was employed to evaluate the quality of raw and mapped snRNA-seq data. We removed empty droplets and droplets with high ambient RNA using DIEM v.2.4.0.^40^ The default parameters were employed, except for using droplets of UMI < 500 as debris and *k* = 50 for the k-means clustering step. We then removed clusters containing highly contaminated droplets based on low average number of unique genes detected (nFeatures), high number of mitochondrial and ribosomal genes as top expressed features, low average UMIs, and high percentage of reads mapped to the mitochondrial genome (%mito). Next, we removed low-quality droplets with nFeatures ≤ 200, UMI ≤ 500, spliced RNA ≥ 75%, and %mito ≥ 10 using Seurat v.4.3.0.1.^41^ We also log-normalized and scaled the counts to a mean of 0 and variance of 1, performed principal component analysis (PCA), and clustered nuclei using the standard Louvain algorithm, with the first 30 PCs and a clustering resolution of 0.5. We removed contaminated counts for the remaining nuclei employing DecontX^45^ using the previously removed low-quality nuclei as a background and Seurat cluster assignment as ‘z,’ and then removed additional low-quality nuclei with UMI ≤ 500, UMI ≥ 30,000, %mito ≥ 10, and nFeatures ≤ 200. To identify the individual of origin for each nucleus, we employed demuxlet from popscle software tool^42^ using imputed genotype data and --min-MQ 30. We only used nuclei classified as singlets and assigned the best-matching participant to each nucleus. Remaining doublets were identified and further removed using DoubletFinder.^46^ We performed pN-pK parameter sweeps to select a pN of 0.25 and the most optimal pK value to maximize, for each batch, the mean-variance normalized bimodality coefficient and obtain a predicted number of doublets that is required for DoubletFinder.^46^

We merged the remaining droplets from all of the batches and subset the nuclei for those that originated from samples collected during bariatric surgery (*n* = 68) using Seurat v.4.3.0.1.^41^ We kept the genes with at least 3 raw counts in at least 3 nuclei^45^ and performed normalization of the gene counts, identification of variable genes, scaling, and PCA, as described above. To account for batch effects, we employed Harmony v.1.0.3^47^ and integrated on batch, and then clustered the nuclei with a standard Louvain algorithm with the first 30 reductions from Harmony and a clustering resolution of 0.5. SingleR v.1.8.1^43^ was used to perform cell-type annotation, as described above, using the SAT snRNA-seq and single-cell RNA-seq data from Emont et al.^48^ as an adipose tissue atlas reference.

### Identification of unique cell-type marker genes in KOBS

To identify unique adipocyte marker genes in the KOBS SAT snRNA-seq data, we used FindAllMarkers from Seurat v.4.0.3.^49^^,^^50^ Accordingly, we first determined the cell-type marker genes for each cell type using default parameters with only.pos = TRUE and logfc.threshold = 0.25.^48^ Genes of each cell type with a Bonferroni-corrected *p* < 0.05 were considered to be marker genes for that particular cell type. We then removed the adipocyte marker genes that were also marker genes for other cell types to obtain unique adipocyte marker genes.

### SAT bulk RNA-seq data from the BMI-discordant MZ twin cohort

The existing SAT bulk RNA-sequencing data in the BMI-discordant MZ twin cohort (*n* = 88) were generated as described previously.^25^^,^^26^^,^^27^^,^^28^^,^^29^ Briefly, paired end 75-bp reads were sequenced using the Illumina HiSeq2000 platform^25^^,^^26^ with an average sequence depth of 40–50 M, and then aligned using STAR^38^ in two-pass mode to the hg38 human reference genome with ENSEMBL v.104 annotations, as described previously.^23^^,^^24^ We used featureCounts^51^ to obtain gene counts and quality control metrics were obtained using Picard (http://broadinstitute.github.io/picard/).

### Adipocyte size measurements in the BMI-discordant MZ twin cohort

We used existing adipocyte size measurements from the BMI-discordant MZ twin cohort (*n* = 88)^26^ in our analysis. As described previously,^26^ fat cell measurements were obtained from SAT biopsies by taking photographs of the fat cells using a light microscope (Zeiss, Axioplan2) at ×50 magnification using a custom algorithm for ImageJ^26^ to automatically infer fat cell diameters.

### Prioritizing adipose cell types based on enriched heritability of abdominal obesity

We used cell type expression-specific integration for complex traits (CELLECT) v.1.3.0 to identify SAT cell types with enriched heritability of obesity-related phenotypes.^52^ To compute expression specificity estimates of genes for each cell type, we used the following three SAT snRNA-seq cohorts: CRYO-Twin (*n* = 15),^14^ KOBS (*n* = 8), and RYSA (*n* = 68). We calculated expression specificity estimates, which consist of average expression of each gene for each cell type in the three cohorts, using CELLEX^52^ on these single-nucleus SAT expression data. We used metabolic dysfunction-associated steatotic liver disease (MASLD) GWAS summary statistics from the UK Biobank,^53^ GIANT, and UK Biobank waist-hip ratio adjusted for BMI (WHRadjBMI) GWAS summary statistics,^11^ and T2D GWAS summary statistics from the Pan-UK Biobank phenotype manifest^54^ to perform stratified linkage disequilibrium score regression (S-LDSC) and quantify the heritability enrichment of the GWAS traits for each cell type. As WHRadjBMI is a commonly used surrogate for abdominal obesity,^2^^,^^3^^,^^4^ we refer to the WHRadjBMI GWAS as the abdominal obesity GWAS.

### Geographic distributions of allele frequencies and F_ST_ of the *TBX15* abdominal obesity GWAS variant rs10494217 using UK Biobank

To identify differences in allele frequencies across populations, we used genotype data from the UKBiobank.^18^ We calculated fixation index, F_ST_, to quantify how much the allele frequency of rs10494217 differs in Finland compared to other geographic locations. We used UKB genotype data from different populations that were determined by the “country of birth” labels (Fields 1647 and 20115) of each participant. To calculate the allele frequency and F_ST_ of rs10494217, we assessed populations with a sample size greater than 30 (*n* = 107 populations). We used the vcftools^55^ command weir-fst-pop to calculate the per-site pairwise F_ST_ between the Finns and these other populations. To assess the F_ST_ of rs10494217 per UKB population, we compared the observed F_ST_ of rs10494217 to the empirical F_ST_ distribution between METSIM and the UKB population for the 10,000 SNPs with the closest allele frequencies to rs10494217 in METSIM. We computed an empirical *p* value for each population based on the proportion of SNPs that had an F_ST_ greater than that of rs10494217.

To further evaluate the F_ST_ results, we also examined the pairwise F_ST_ for rs10494217 between Denmark, England, Finland, Italy, Norway, Spain, Sweden, Switzerland, and the remaining UKB populations with *n* > 30 as follows. We derived the empirical *p* value per population pair by comparing the F_ST_ of rs10494217 to the corresponding empirical F_ST_ distributions of all genome-wide SNPs with MAF > 0.05 in METSIM. We also calculated the empirical *p* value based on the distribution of the 10,000 SNPs with allele frequencies closest to rs10494217 in the METSIM cohort, similar as we did between METSIM and the UKB populations above, for all pairs of populations in UKB with *n* > 30.

### Investigating LD structure around the *TBX15* abdominal obesity GWAS variant

To study the LD structure around rs10494217 in Finns and other populations, we used phased, imputed METSIM genotype data and similarly phased, imputed UK Biobank genotype data. Briefly, similarly as in METSIM, we used KING v.2.3.2 with --unrelated --degree 3^32^ to first identify the 3^rd^-degree unrelated males of British origin in UKB. We randomly selected 6,738 unrelated British males from UKB, to match the samples size of unrelated men in METSIM, and then lifted over their genotype data from build hg19 to hg38 using Picard Tools (http://broadinstitute.github.io/picard/). Eagle v.2.4,^35^ in reference free mode with default parameters, was employed to phase the data. We calculated and visualized the pairwise LD for all variants within a window between 118,916,565 and 118,942,565 bp on chromosome 1 with MAF > 0.05 using HaploView –r2.^56^

### Phylogenetic generalized least squares analysis

We conducted a phylogenetic generalized least squares (PGLS) analysis to predict allele frequencies of rs10494217 from ancestry, latitude, and average temperature, while correcting for population structure. Ancestry was defined using the population classifications provided by the 1000 Genomes Project. We used the average annual temperatures and latitudes for 22 populations from Phase 3 of the 1000 Genomes Project,^57^ and the neighborhood joining tree, computed from pairwise F_ST_ estimates of 61 randomly chosen individuals per population, all of which were previously computed.^58^ As in Key et al.,^58^ we modeled the allele frequency of rs10494217 in each down-sampled population dataset of the 1000 Genomes Project in a null model, consisting of only the population structure from the neighborhood joining tree, and in a full model, consisting of the population structure, latitude, and annual mean temperature. After verifying the stability of the full model with a leave-one-out approach of populations and assessing normality of the residuals, a likelihood ratio test was performed to evaluate goodness-of-fit for the model. We then used a multi-modal inference analysis to compare the null model with all possible models from the two predictors, using Akaike weights of each model to assess the model confidence and assign weights to the predictors, as previously done.^58^

### Evaluating extended haplotype homozygosity around rs10494217 in Finns

We searched for putative selection of rs10494217 in Finns using the METSIM (*n* = 6,738) genotype data of individuals unrelated to the third degree. Extended haploytype homozygosity (EHH) is computed by determining the probability that two randomly chosen chromosomes with the same allele at the focal SNP lie on identical genomic regions.^59^^,^^60^ EHH is calculated starting at 1 at the focal SNP and decays to 0 with increasing distance away from the focal SNP.^59^^,^^60^ We used Hapbin^61^ to derive the EHH surrounding rs10494217 to determine the distance the haplotype homozygosity continues from rs10494217. Then, we used Hapbin^61^ to compute the integral of EHH, a powerful statistic known as iHS,^62^ for all SNPs on chromosome 1 and to then determine how selection of rs10494217 compares to the empirical distribution. We employed the genetic map downloaded from Eagle using the GRCh38 reference to overlap with the set of genotyped and imputed SNPs in our study to obtain genetic distance. We used the default parameters for all calculations, and a minimum MAF of 0.05. Additionally, iHS is standardized in allele frequency bins^61^ using SNPs on chromosome 1. We calculated the percentile in which the rs10494217 standardized iHS value falls compared to other chromosome 1 SNPs. We also compared the standardized iHS value of rs10494217 to the genome-wide SNPs with MAF > 0.05. Finally, we compared the standardized iHS value of rs10494217 to the genome-wide SNPs with a MAF between 0.25 and 0.30.

### Quality control and phasing of UKB genotype data for iHS calculations

We computed the iHS statistic^62^ for rs10494217 for all UKB individuals from Sweden (*n* = 214), Denmark (*n* = 229), Norway (*n* = 133), Italy (*n* = 812), as well as 6,738 randomly selected unrelated males of British origin from UKB. We first identified the 3rd-degree unrelated individuals from each population using KING v.2.3.2^32^ with --unrelated --degree 3. We then lifted over the genotype data from chromosome 1 of the unrelated individuals from build hg19 to hg38 using Picard Tools (http://broadinstitute.github.io/picard/), and phased the hg38 data using Eagle v.2.4^35^ in a reference free mode with default parameters. Finally, we performed the iHS calculations and determined the percentile into which rs10494217 falls when compared to the other SNPs in each population, similarly as described above for the Finns.

### Analysis of unique adipocyte marker gene expression based on the risk allele status of the *TBX15* abdominal obesity variant rs10494217

To identify potential downstream *trans* effects of the abdominal obesity GWAS variant, rs10494217, on adipocyte expression, we performed a risk allele status analysis in adipocytes in the KOBS snRNA-seq data between the individuals with the T risk allele (*n* = 4) and without the T risk allele (*n* = 4) of rs10494217. We defined the individuals that have the risk allele as individuals with at least one copy of the T allele, and the individuals that do not harbor the risk allele as homozygous for the G allele. We ran a Wilcoxon rank-sum test on the KOBS adipocyte snRNA-seq expression data using the FindMarkers function in Seurat v.4.3.0^50^ with a log fold-change (logFC) threshold of ≥0 and tested only the unique adipocyte marker genes in KOBS for a risk allele status-based expression effects using a Bonferroni adjusted *p*_adj_ < 0.05. Our rationale to use a logFC threshold of ≥0 is that we are examining subtle differences within the same cell type. To replicate these findings in adipocytes from the RYSA SAT snRNA-seq data, we performed the same risk allele status analysis using the genes found to be affected by the risk allele status in KOBS and similarly tested them between the individuals with (*n* = 36) and without (*n* = 32) the abdominal obesity risk allele T of the rs10494217 in the RYSA cohort. In the replication analysis, we applied a Bonferroni correction for multiple testing and considered the genes with a *p*_adj_ < 0.05 and a log fold-change in the same direction as in KOBS to be replicated in RYSA. The unique adipocyte marker genes with replicated risk allele associations are referred to as *trans* genes.

As *TBX15* has previously shown sex-specific effects on SAT expression,^14^ we also performed the risk allele status analysis separately in males (*n* = 19) and females (*n* = 49) for the 13 *trans* genes using adipocyte expression from the RYSA SAT snRNA-seq data. We ran a Wilcoxon rank-sum test employing the FindMarkers function in Seurat^50^ on the *trans* genes using a logFC threshold of ≥0 separately in males and females.

### Module score calculations with the *trans* genes using SAT snRNA-seq data in KOBS and RYSA

To determine whether the average expression of the *trans* genes was significantly different between the individuals with and without the risk allele of rs10494217, we used the AddModuleScore function in Seurat v.4.3.0^50^ with the *trans* genes as input features and employing the default parameters. We calculated the module scores in all nuclei labeled as adipocytes, and then performed a non-paired Wilcoxon text to compare the module scores between the cells from those that do and do not have the risk allele.

### Identification of genes with pairwise correlated adipocyte expression

To identify whether any of the 4 lncRNA *trans* genes were correlated with their nearby genes, we calculated pairwise gene-gene Spearman correlations using adipocyte expression from the SAT snRNA-seq data in RYSA (*n* = 68). The genes within 1 MB upstream and downstream of the transcription start and stop sites of each lncRNA were selected for these gene-gene correlation analyses. We used adipocyte pseudobulk expression data that we TMM normalized, adjusted for the number of nuclei, age, and sex, and inverse normal transformed. We then performed pairwise Spearman correlations between the normalized adipocyte expression of the genes.

### Correlating adipose gene expression with adipocyte size measurements

We correlated gene expression of the 10 genes around the lncRNA *AC002066.1* with adipocyte size measurements. For these correlation analyses, we used the SAT bulk RNA-seq and adipocyte diameter data from the Finnish BMI-discordant MZ twin cohort. We first computed TPMs in the SAT bulk RNA-seq data, then corrected the data for RIN, 3′ bias, percent of uniquely mapped reads, and the percent of mitochondrial reads, and finally applied an inverse normal transformation. The adipocyte diameter was also inverse normal transformed to account for outliers. We divided the BMI-discordant twin pairs into the higher (*n* = 45) or lower (*n* = 43) BMI twin groups, then performed Spearman correlations between gene expression and fat cell measurements separately for each group. The *p* values were adjusted for multiple testing using Bonferroni (*p*_adj_ < 0.05).

## Results

### Overview of the study design

**Figure 1:**
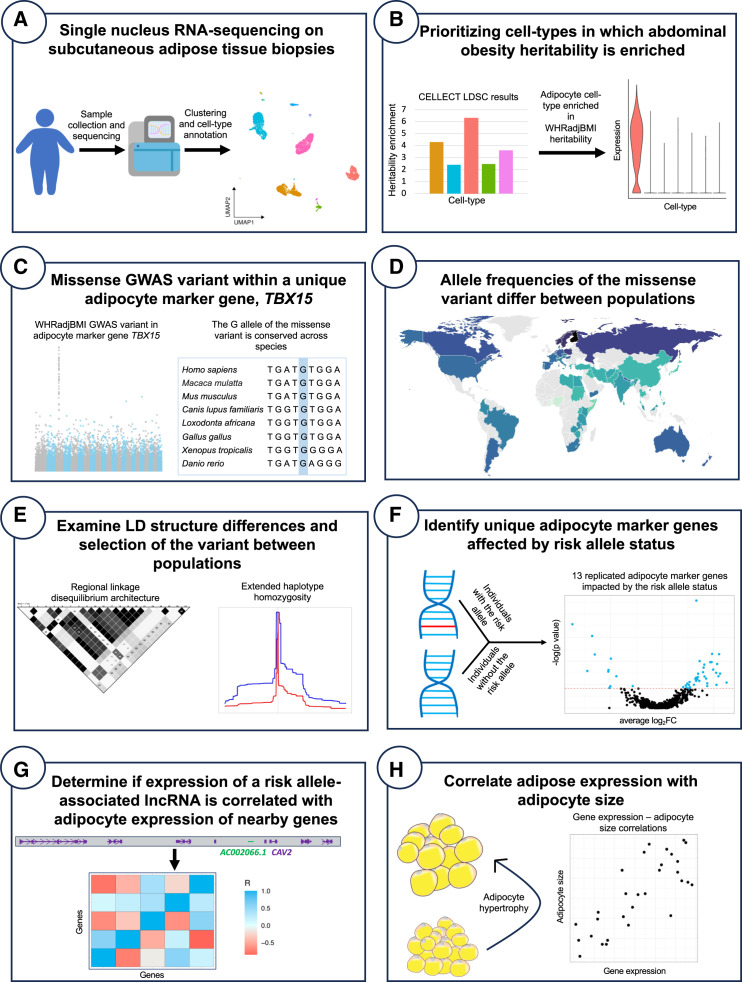
A stepwise overview of our study design investigating an abdominal obesity GWAS missense variant in *TBX15* for signals of selection and adipose cell-type expression changes (A) We generated subcutaneous adipose tissue (SAT) single nucleus RNA-sequencing (snRNA-seq) data from biopsies from Finnish individuals with obesity undergoing bariatric surgery, then clustered and annotated the cell types. (B) We found the SAT adipocyte cell type to be enriched for the heritability of abdominal obesity. (C) We focused on a specific unique adipocyte marker gene containing an abdominal obesity GWAS missense variant. (D) We found a north-to-south allele frequency gradient for the missense variant. (E) We examined the LD architecture around the variant and investigated the variant for signals of selection. (F) We compared adipocyte expression of unique adipocyte marker genes between the individuals that do and do not harbor the risk allele of the abdominal obesity GWAS missense variant. (G) We investigated whether adipocyte expression of any of the risk allele-associated long non-coding RNAs (lncRNAs) is correlated with expression of nearby genes, suggesting a further downstream regulatory mechanism of *TBX15* in *trans*. (H) We correlated adipose expression of a risk allele-associated lncRNA (and regional genes correlated with it) with adipocyte size in an independent cohort to search for effects on adipocyte hypertrophy.

The mechanisms of action by which GWAS variants contribute to phenotypic changes are unknown for the vast majority of GWAS variants. To identify how abdominal obesity GWAS variants affect cell-type gene expression and ultimately contribute to abdominal obesity and related comorbidities, we leveraged SAT snRNA-seq data from three Finnish cohorts, KOBS, CRYO-Twin, and RYSA (see methods). A schematic overview of the study design is shown in Figure 1. Briefly, we began by searching for SAT cell types, in which expression of genes is enriched for heritability of obesity traits, finding that the adipocyte cell type shows significantly enriched heritability for WHRadjBMI. As WHRadjBMI is a well-established proxy for abdominal obesity,^2^^,^^3^^,^^4^ we will refer to the WHRadjBMI GWAS as the abdominal obesity GWAS. As the SAT snRNA-seq data allow investigation of risk allele-based expression of coding variants at the cell-type resolution, we focused on abdominal obesity GWAS SNPs within the coding regions of the unique adipocyte marker genes. We found only one missense variant residing in the T-Box Transcription Factor 15 (*TBX15*) gene, a unique adipocyte marker gene that encodes a transcription factor (TF) that has been shown to regulate a SAT bulk co-expression network associated with abdominal obesity.^14^ Our downstream analyses then centered around this abdominal obesity GWAS missense variant, rs10494217, which is conserved across species (Figure 1) and predicted to be potentially deleterious. We observed a high allele frequency of rs10494217 in Finns, a genetically isolated population^63^ when compared to other populations, suggesting putative selection. To address this possibility, we performed selection analyses using genotype data from Finns and 106 other populations. Our goal was to determine whether the abdominal obesity risk allele T of rs10494217 is positively selected in the Finns. Finally, since TBX15 is a TF, we analyzed whether downstream SAT adipocyte expression of genes is impacted by the T risk allele of rs10494217 in *trans* (Figure 1).

### SAT adipocytes are enriched for abdominal obesity heritability

To identify SAT cell types implicated in heritability of obesity and related comorbidities, we used the CELLECT tool^52^ with SAT snRNA-seq data. Our goal was to test whether heritability of obesity traits is enriched in *cis r*egions of the genes that are expressed in specific SAT cell types. For these analyses, we used GWAS summary statistics from previously conducted extensive GWASs of abdominal obesity,^11^ T2D,^54^ and MASLD.^53^ We observed a significant (Bonferroni-adjusted *p*_adj_ < 0.05) enrichment of abdominal obesity GWAS variants in the adipocyte cell type consistently in all three cohorts (*p*_KOBS_adj_ = 1.16 × 10^−3^, *p*_CRYO_adj_ = 9.40 × 10^−4^, *p*_RYSA_adj_ = 5.70 × 10^−5^), whereas no other cell types were significantly enriched for heritability of any of the three assessed outcomes (Table S1). Given that WHRadjBMI has previously been established as a surrogate for abdominal obesity,^2^^,^^4^ these consistent results obtained in three independent SAT snRNA-seq cohorts suggest that the adipocyte cell type plays an important role in abdominal obesity.

### Identification of an abdominal obesity GWAS missense variant within a unique adipocyte marker gene

Next, we aimed to trace this genetic abdominal obesity enrichment back to key functional drivers within SAT. We narrowed our analysis to coding GWAS variants as we can assess their risk allele effects on cell-type gene expression in the snRNA-seq data. We specifically focused on abdominal obesity GWAS variants within the coding regions of the unique adipocyte marker genes due to their high cell-type expression and the heritability enrichment of abdominal obesity in adipocyte-expressed genes that we detected using CELLECT (Table S1). Intriguingly, we found that *TBX15*, which is a unique adipocyte marker gene in the snRNA-seq data both from KOBS and RYSA, contained one common missense abdominal obesity GWAS SNP, rs10494217. *TBX15* is not a unique adipocyte marker gene in the CRYO cohort, likely due to BMI effects as participants in CRYO-Twin have a significantly lower BMI than the participants of the bariatric surgery obesity cohorts, KOBS and RYSA (mean BMI ± SD is 37.9 ± 1.65 in KOBS; 43.1 ± 5.41 in RYSA; and 31.45 ± 5.42 in CRYO, *p*_KOBS-CRYO_ = 7.75 × 10^−5^, *p*_RYSA-CRYO_ = 1.30 × 10^−8^), suggesting that the adipocyte expression of *TBX15* is upregulated by obesity.

**Figure 2:**
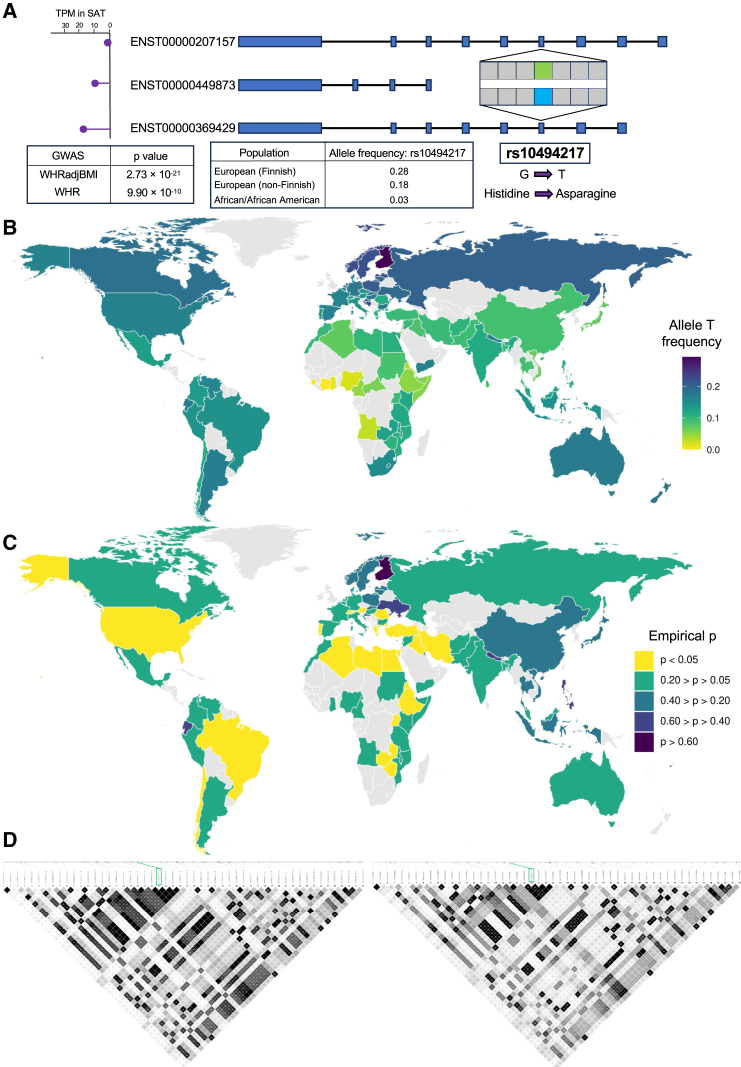
Allele frequencies and regional linkage disequilibrium differ between Finns and other populations for rs10494217, the missense variant present in two transcripts of *TBX15* (A) A schematic overview showing the location of the missense variant, rs10494217, within *TBX15* and expression of the three *TBX15* transcripts in SAT using GTEx.^64^ Allele frequencies shown for rs10494217 are from the gnomAD database.^69^ GWAS association *p* values for the traits are obtained from the GIANT-UKB meta-analysis^11^ for both waist-hip ratio (WHR) and abdominal obesity, measured by waist-hip ratio adjusted for body mass index (WHRadjBMI). (B) Allele frequencies of the *TBX15* missense variant based on the country of origin among the UK Biobank (UKB) participants. Allele frequency is shown for populations with *n* > 30. (C) We show empirical *p* values for the F_ST_ of rs10494217, calculated based on the proportion of SNPs with greater F_ST_ than rs10494217. A total of 10,000 SNPs were used for the empirical distribution, chosen based on their similar minor allele frequencies to rs10494217 in the METSIM cohort. (D) LD (linkage disequilibrium) architecture surrounding the missense variant rs10494217 (labeled in the green box) in the Finns (left, *n* = 6,738 in METSIM) and Brits (right, *n* = 6,738 in UKB) for the region on chromosome 1 between 118,916,565 and 118,942,565 bp in GRCh38. The same SNPs were included for both cohorts. Maps were constructed using HaploView^56^ and LD values are shown in R^2^.

Next, we focused on this common missense abdominal obesity GWAS SNP, rs10494217, in *TBX15*. The variant is also a known GWAS variant for a related trait, WHR (Figure 2A), and present in two of the three *TBX15* transcripts, ENST00000369429 and ENST00000207157 (Figure 2A). We then looked at the expression of each of these transcripts using GTEx v.8 data^64^ and observed that one of the transcripts, ENST00000369429, containing the missense variant rs10494217 is the highest expressed *TBX15* transcript in SAT (Figure 2A). Additionally, this SNP causes a change in the amino acids in TBX15 from histidine, a positively charged amino acid, to asparagine, a polar amino acid (Figure 2A). When using the Variant Effect Predictor (VEP) tool,^65^ we found that rs10494217 is predicted to be deleterious in one transcript, ENST00000369429, and deleterious with low confidence in the other transcript, ENST00000207157, by the Sorting Intolerant from Tolerant (SIFT) database.^66^ The Protein Analysis Through Evolutionary Relationships (PANTHER) database predicts rs10494217 to be “probably damaging” in both transcripts as well.^67^ Finally, the CADD-SV score is 25.7 from the Combined Annotation Dependent Depletion (CADD) database.^68^ In line with these results, we also found by direct sequence comparisons in other species that the variant is highly conserved across species (Figure 1). Taken together, the missense variant rs10494217 could alter the structure or function of TBX15, and accordingly affect its binding to the downstream target genes.

### The allele frequency of rs10494217 differs between populations

To characterize the prevalence of the rs10494217 abdominal obesity risk allele at the population level, we used the gnomAD browser.^69^ Interestingly, we first observed an allele frequency difference between Finns and non-Finn Europeans (MAF of 0.28 in Finns versus 0.18 in non-Finn Europeans) (Figure 2A). Moreover, we found the allele frequency of the abdominal obesity risk allele T to drop to 0.03 in African/African American populations (Figure 2A). We explored this with more detail in Figure 2B, showing the allele frequency of the T risk allele of rs10494217 in 107 different populations (*n* > 30) using UKB. We found that the allele frequency of the T allele was lower in populations closer to the equator and higher with increasing distance away from the equator, with Finns having the highest MAF (Figure 2B; Table S2), suggesting a potential descending allele frequency gradient from north to the equator.

Having observed this 10× difference in MAFs, we next examined the population differences using fixation index, F_ST_ (see methods). We calculated pairwise F_ST_ between the Finns using METSIM and each of the 107 populations in the UKB that contained sample sizes of over 30 (Figure S1). To determine whether the F_ST_ values observed for rs10494217 were significant compared to other SNPs in the genome, we ranked the F_ST_ relative to the empirical F_ST_ distribution of 10,000 SNPs with allele frequencies closest to rs10494217 in the METSIM Finns. We found that the F_ST_ of rs10494217 between the METSIM Finns and the test population in UKB was in the top 5% of the F_ST_ values (*p* < 0.05) among the 10,000 tested SNPs in 25 populations of the 107 (23.4%) UKB populations with *n* > 30 (Figure 2C; Table S2). Noteworthy, many of these significant F_ST_ values were found between individuals in Finland and countries with warmer climates, such as Uganda (*p* = 7.4 × 10^−4^), Libya (*p* = 0.020), and Egypt (*p* = 0.022) (Figure 2C; Table S2).

We also ran additional F_ST_ analyses in UKB, where we assessed the F_ST_ of rs10494217 between various UKB countries. We calculated F_ST_ between all pairs of UKB populations with *n* > 30 relative to the corresponding empirical F_ST_ distributions of the 10,000 SNPs with allele frequencies closest to rs10494217 in the METSIM cohort, similarly as we did between METSIM and UKB above. We also calculated empirical *p* values using all genome-wide SNPs with a MAF > 0.05 (see methods) for the F_ST_ between the UKB populations of Finland, Switzerland, Norway, Sweden, England, Italy, Spain, and Denmark and the remaining UKB populations with *n* > 30. In these analyses, we found that the Finns show the strongest significant F_ST_ values in comparisons with other countries latitudinally (*n* = 57 populations significant in both analyses) (Tables S3 and S4). Regarding the non-Finnish European countries, we observed significant F_ST_ values between the other northern European countries, Sweden and Norway, and southern, warmer countries, similarly as with the Finns, whereas the central and southern European countries do not show these significant F_ST_ values latitudinally (Tables S3 and S4). Taken together, we found evidence for a north-south difference among the global allele frequencies with the abdominal obesity GWAS *TBX15* missense variant rs10494217, where a higher frequency of the T allele, the risk allele for abdominal obesity, appears in more northern countries with colder climates than in the southern warmer countries, in line with the previous animal studies reporting a thermogenic effect of Tbx15.^12^^,^^13^

### The LD structure of rs10494217 differs between Finns and Brits

As we noticed the frequency of the T allele of rs10494217 to be dissimilar between the Finns and non-Finn Europeans, we hypothesized that the local linkage disequilibrium (LD) structure may also differ between the two populations. Visualizing the LD structure around rs10494217 in the Finns using METSIM and in Brits using UKB, we observed a stronger LD structure in the region surrounding rs10494217 in the Finns than Brits (Figures 2D and S2A). We quantified this difference in LD by performing a Wilcoxon rank-sum test comparing the R^2^ values for the LD between rs10494217 and SNPs within 1 Mb from rs10494217 on each side between the Finns and Brits and found the difference to be significant (*p* = 1.31 × 10^−41^) (Figures S2A and S2B).

### The allele frequency of rs10494217 differs along a latitudinal cline

**Figure 3:**
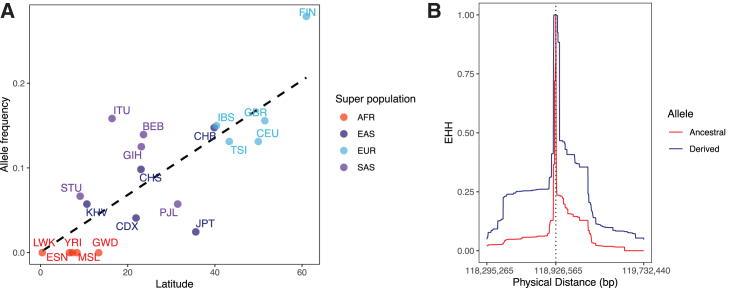
The risk allele T of the abdominal obesity GWAS SNP, rs10494217, differs in frequency on a latitudinal cline and is selected for in the Finns (A) Correlation of the allele frequency of the T risk allele of rs10494217 with latitude using the populations of the 1000 Genomes Project. The trend line depicts the results of the PGLS analysis. Superpopulations based on the classifications by the 1000 Genomes Project are shown as follows: AFR (African), EAS (East Asian), EUR (European), and SAS (South Asian). The populations are represented as follows: BEB (Bengali in Bangladesh), CDX (Chinese Dai in Xishuangbanna, China), CEU (Utah residents, USA, with Northern and Western European ancestry), CHB (Han Chinese in Beijing, China), CHS (Southern Han Chinese), ESN (Esan in Nigeria), FIN (Finnish from Finland), GBR (British from England and Scotland), GIH (Gujarati Indians in Houston, USA), GWD (Gambian [Mandinka] in Western Divisions in Gambia), IBS (Iberian populations in Spain), ITU (Indian Telugu in the UK), JPT (Japanese in Tokyo, Japan), KHV (Kinh in Ho Chi Minh City, Vietnam), LWK (Luhya in Webuye, Kenya), MSL (Mende in Sierra Leone), PJL (Punjabi in Lahore, Pakistan), STU (Sri Lankan Tamil in the UK), TSI (Toscani in Italia), and YRI (Yoruba in Ibadan, Nigeria). (B) Extended haplotype homozygosity (EHH) is shown for rs10494217 in the Finns (*n* = 6,738 in METSIM). The derived allele indicates the T risk allele for abdominal obesity and the ancestral allele indicates the G allele. The vertical dashed line depicts the genomic location of rs10494217. The genomic coordinates shown refer to the GRCh38 genome build.

Because the population of Finland has been subjected to bottlenecks in the past,^16^ it is possible that the unique population structure of Finland could be responsible for the elevated allele frequency of rs10494217. To address whether our results are driven by demographic processes or selection, we checked whether latitude, temperature, and shared ancestry predict the observed allele frequency of rs10494217. We used a PGLS test for this analysis with data from the 1000 Genomes Project (see methods), similarly as was performed for *TRPM8* in a previous latitudinal selection study.^58^ In this PGLS approach, where we are modeling the allele frequencies of multiple countries, the bottlenecks that Finland has undergone are not a major factor in the analysis. We found that the full model, including the latitude and temperature as predictor variables, significantly predicts the allele frequency of rs10494217 (*p* = 1.58 × 10^−5^), whereas the null model, including ancestry information only, does not significantly predict the allele frequency of rs10494217. When assessing each predictor variable independently in the full model, we found significant support for the model with latitude (*p =* 0.0035, Figure 3A). Thus, latitude is a better predictor of the frequency of the T abdominal obesity risk allele than shared ancestry, supporting our hypothesis of latitude-based selection. Similarly as in Key at al.,^58^ we found that the annual temperature is not a significant predictor alone (*p* > 0.05), which could be attributed to the instability of the annual temperature used in this analysis and simplicity of the average annual temperature when compared to actual seasonal temperatures. Overall, the PGLS analysis supports a significant latitudinal cline of the allele frequency for the abdominal obesity risk allele T of rs10494217.

### Selection for rs10494217 in Finns

The observed significance of F_ST_ and latitudinal cline of allele frequency of rs10494217 in Finns alludes to the potential for positive selection. This selection could be specific to Finns, given the clear differences in the allele frequencies, LD structure in Finns compared to other populations, and the known population history of Finns as a genetic isolate. To address this possibility, we used extended haplotype homozygosity (EHH) to identify signatures of recent positive selection in the genome. EHH is based on the concept that an allele under positive selection increases in frequency faster than recombination can break down the haplotype on which it resides, resulting in unusually long haplotypes carrying one allele as opposed to the other. We used genotype data from METSIM (*n* = 6,738) to measure the haplotype breakdown for the major and minor allele of rs10494217. We found that the haplotype carrying the derived allele, i.e., the T allele, has greater EHH than the haplotype carrying the ancestral allele. Specifically, in Finns the EHH (measured until EHH < 0.05) for the derived allele extends 319 kilobases farther than the EHH of the ancestral allele in one direction and 500 kilobases farther than the EHH of the ancestral allele in the other direction (Figure 3B; Table S5). Next, we calculated the iHS, used as a quantitative metric for selection, for all SNPs on chromosome 1 with MAF > 0.05. Previous studies have considered an |iHS| ≥ 2 as a signature of recent or ongoing selection.^70^ The iHS for rs10494217 is −2.02 before standardization, then −2.03 after standardizing in allele frequency bins across chromosome 1. The negative sign implies that the derived allele, the T abdominal obesity risk allele, is being selected for when compared to the ancestral allele. The standardized iHS for rs10494217 is in the 96^th^ percentile for chromosome-wide iHS values (Table S6). We also investigated how rs10494217 compares to the SNPs across all chromosomes with MAF 0.25–0.30, as well as to all SNPs with MAF > 0.05 (for full results, see data and code availability) and found similarly that rs10494217 is in the 96^th^ percentile of the standardized iHS values in both comparisons. Taken together, these results suggest that the T risk allele of rs10494217 is being selected for in Finns.

We hypothesize that the signatures of selection are due to the role of TBX15 in thermogenesis, i.e., in populations residing in climates similar to Finland, the abdominal obesity risk allele T of rs10494217 also has the potential to be selected for. To test this, we ran EHH for rs10494217 using the UKB participants from Sweden, Denmark, Norway, the UK, and Italy (see methods). The results from this analysis are as follows: the standardized iHS of rs10494217 is −1.57 in Sweden, which is in the 88^th^ percentile; −1.36 in Denmark, the 83^rd^ percentile; −1.22 in Norway, the 77^th^ percentile; −1.38 in the UK, the 83^rd^ percentile; and −1.07 in Italy, the 71^st^ percentile, respectively. These north-to-south decreasing trends in iHS results appear to align with the results in Finns; however, we observed a significant selective signature, quantified by |iHS| ≥ 2, only in Finland. Taken together, these results support our hypothesis of selection of the T risk allele of rs10494217 in Finns.

### Adipocyte expression of 13 unique adipocyte marker genes is affected by the risk allele status of the rs10494217 abdominal obesity GWAS variant

**Figure 4:**
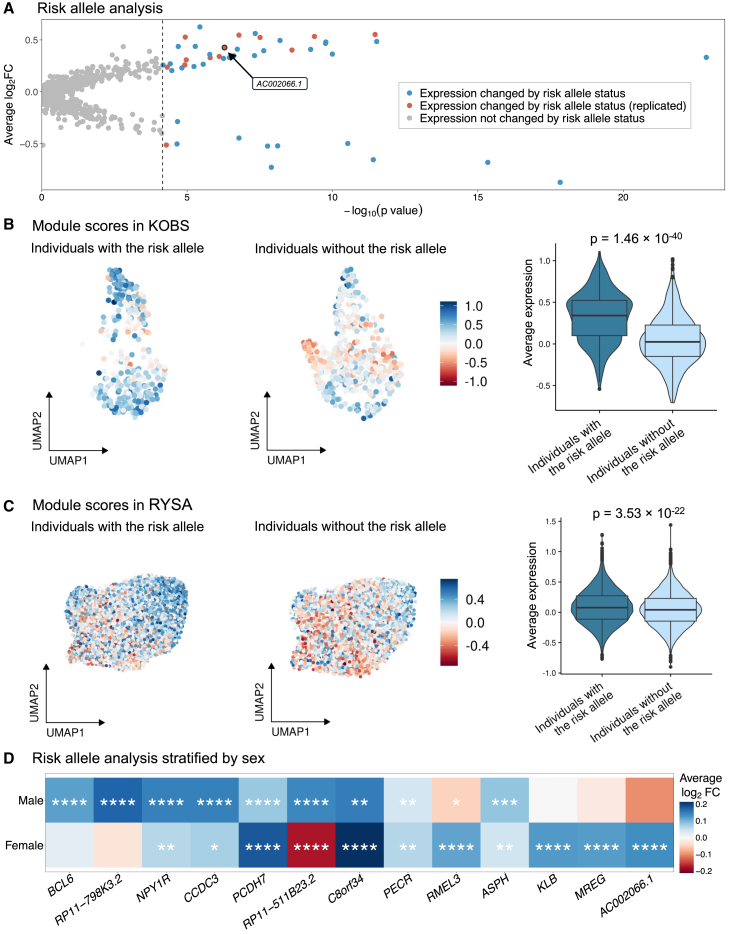
The effect of abdominal obesity variant rs10494217 on adipocyte expression replicated for 13 unique adipocyte marker genes (A) Analysis comparing adipocyte expression of unique adipocyte marker genes by the risk allele status of the abdominal obesity GWAS variant rs10494217. Average log-fold change and the Wilcoxon *p* values are shown for KOBS (*n* = 8), and we show in red the 13 genes replicated in RYSA (*n* = 68). (B and C) Module scores based on average adipocyte expression of the 12 genes upregulated in the individuals that harbor the risk allele in adipocytes in KOBS (total *n* = 8; 4 individuals with the risk allele) (B) and RYSA (total *n* = 68; 36 individuals with the risk allele) (C). We label the Wilcoxon *p* value comparing the module scores between the two groups. (D) Adipocyte expression differences by the risk allele status, shown separately in males (total *n* = 19; 11 individuals with the risk allele) and females (total *n* = 49; 25 individuals with the risk allele) of the RYSA cohort. Significance thresholds for the Bonferroni adjusted Wilcoxon *p* values: ^∗^*p*_adj_ < 0.05, ^∗∗^*p*_adj_ < 0.01, ^∗∗∗^*p*_adj_ < 0.001, and ^∗∗∗∗^*p*_adj_ < 0.0001.

As TBX15 is a TF, a change in its amino acid content may affect its function and thus transcription of its downstream target genes.^71^ Therefore, we conducted a risk allele status analysis using the individuals that do and do not harbor the risk allele of the rs10494217 abdominal obesity risk allele to identify potential target genes with adipocyte expression patterns linked to the rs10494217 risk allele. Since we found the adipocyte cell type to be enriched for abdominal obesity heritability using the CELLECT tool^52^ and *TBX15* to be a unique adipocyte marker gene consistently in the obese Finnish cohorts, we focused our analysis on unique adipocyte marker genes and adipocyte expression. This choice was further supported by our observation that the unique adipocyte marker genes are significantly (FDR < 0.05) enriched for pathways relevant for adipocyte function (Table S7) when compared to the same number of genes randomly selected among all adipocyte-expressed genes, which show no significant pathway enrichments (FDR = 1). We first tested unique adipocyte marker genes (*n* = 722) in adipocytes from the KOBS SAT snRNA-seq data between the individuals with the risk allele (*n* = 4) and those without the risk allele (*n* = 4) of the rs10494217 abdominal obesity risk allele (see methods). We identified 47 unique adipocyte marker genes that affected by the risk allele status passing correction for multiple testing using Bonferroni-adjusted *p* < 0.05 (Figure 4A; Table S8). Next, to replicate our findings, we tested these 47 unique adipocyte marker genes for replication between the individuals with (*n* = 36) and without (*n* = 32) the abdominal obesity risk allele T of rs10494217 in the SAT adipocyte snRNA-seq data from an independent bariatric surgery cohort, RYSA, using Bonferroni correction for multiple testing. Ultimately, we found 13 risk allele status-associated unique adipocyte marker genes (Bonferroni-adjusted *p* < 0.05) in the same direction of effect between the individuals with and without the risk allele of the rs10494217 risk allele T in RYSA as in KOBS (Figure 4A; Table S9). We define these 13 replicated genes as *trans* genes.

To further investigate these results, we assessed the average adipocyte expression profiles of these *trans* genes between the individuals with and without the abdominal obesity risk allele, rs10494217, through computing module scores and using a Wilcoxon rank-sum test to verify whether the average adipocyte expression of these genes is affected by rs10494217 in an allele-specific way. We used the 12 *trans* genes, for which the adipocyte expression is upregulated in the individuals that have the T allele of rs10494217, to compute module scores. We found the difference in module scores between the individuals with and without the rs10494217 T risk allele to be highly significant (*p*_KOBS_ = 1.46 × 10^−40^, *p*_RYSA_ = 3.53 × 10^−22^), consistently in both cohorts (Figures 4B and 4C). To further confirm this, we then performed a permutation analysis where we shuffled the risk allele status of the individuals in RYSA and performed Wilcoxon rank-sum tests of the module scores between the two groups. In 10,000 permutations, no combination had a *p* value as significant as the observed *p* value obtained with the 12 *trans* genes (*p*_perm_ ≤ 9.9 × 10^−5^). Taken together, these results indicate that the adipocyte expression of the 13 unique adipocyte marker genes is connected to the rs10494217 abdominal obesity risk allele status.

As *TBX15* has previously been identified to be sex specific,^14^ we also performed the risk allele status analysis separately in males (*n* = 19) and females (*n* = 49) using the RYSA snRNA-seq gene expression data within the adipocyte cell type. We found that five of the thirteen genes were significant in only one sex (Figure 4D; Tables S10 and S11), highlighting the sex specificity of *TBX15*.

### Expression of *trans* lncRNA is correlated with expression of nearby genes

**Figure 5:**
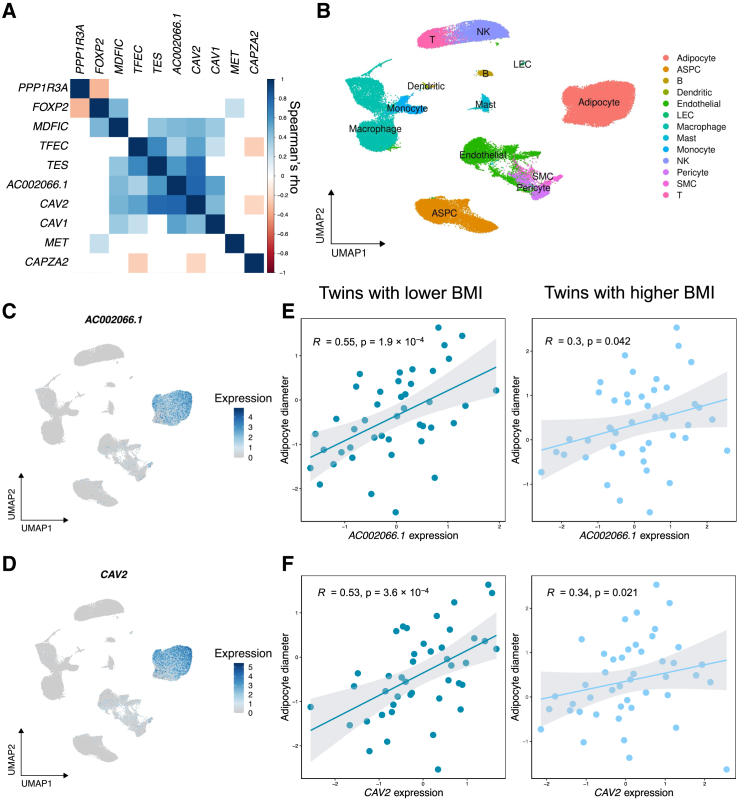
Expression of *trans* long non-coding RNA, *AC002066.1*, is correlated with expression of regional genes, including *CAV2*, and adipocyte size (A) Gene-gene Spearman correlations for the genes in the long non-coding RNA (lncRNA), *AC002066.1*, region using adipocyte pseudobulk in RYSA (*n* = 68). The scale shows Spearman’s rho. White boxes indicate the pairwise correlations that are not nominally significant (*p* > 0.05). (B) Uniform manifold and approximation projection (UMAP) visualization of 67,563 nuclei in the SAT snRNA-seq data from RYSA (*n* = 68) colored by the cell type. The cell types are as follows: ASPC refers to adipose stem and progenitor cells, B to B cells, LEC to lymphatic endothelial cells, NK to natural killer cells, SMC to smooth muscle cells, and T to T cells. (C) UMAP visualization of *AC002066.1* expression in the SAT snRNA-seq data from RYSA (*n* = 68). (D) UMAP visualization of *CAV2* adipocyte expression in the SAT snRNA-seq data from RYSA (*n* = 68). (E) Spearman correlations and *p* values of the correlations between adipose expression in TPMs of *AC0002066.1* and adipocyte diameter using the Finnish BMI-discordant MZ twin cohort. Each twin pair was divided into the lower BMI (*n* = 43) and higher BMI (*n* = 45) groups, and the correlations were performed separately in each group. (F) Spearman correlations and *p* values of the correlations between adipose expression of *CAV2* and adipocyte diameter using the Finnish BMI-discordant MZ twin cohort. Each twin pair was divided into the lower BMI (*n* = 43) and higher BMI (*n* = 45) groups, and the correlations were performed separately in each group.

Four of the 13 *trans* adipocyte marker genes were long non-coding RNAs (lncRNAs): *AC002066.1*, *RMEL3*, *RP11-511B23.2*, and *RP11-798K3.2*. Because lncRNAs have been found to regulate expression of nearby genes,^72^ we next investigated regulation of their regional genes as a possible mechanism for these four lncRNAs. Specifically, we examined the gene-gene correlation patterns for genes near the four lncRNAs in each region surrounding the particular lncRNA (±1 Mb around the transcription start/stop sites of each lncRNA) using the RYSA adipocyte snRNA-seq data (Table S12). We found that adipocyte expression of one lncRNA, *AC002066.1*, the adipocyte expression of which was upregulated in the individuals that have the rs10494217 risk allele, was highly positively correlated with 5 of its regional genes (0.33 < Spearman’s rho < 0.80, *p*_*MDFIC*_ = 4.29 × 10^−5^, *p*_*TFEC*_ = 1.43 × 10^−3^, *p*_*TES*_ = 2.77 × 10^−8^, *p*_*CAV2*_ = 6.68 × 10^−18^, *p*_*CAV1*_ = 6.00 × 10^−6^) among the 10 regional genes tested (Figure 5A; Table S12). As *AC002066.1* is a unique adipocyte marker gene (Figures 5B and 5C), we examined whether its regional genes also have similar expression patterns in SAT cell types. We found *CAV2* to be a unique adipocyte marker gene (Figures 5B and 5D), and then checked its risk allele status result in KOBS, which showed a nominal *p* value of 0.00128 though it did not pass the Bonferroni correction in KOBS (*p*_adj_ > 0.05).

### *trans* gene *AC002066.*1 is positively correlated with adipocyte size

We observed that one of the 5 regional genes in the gene-gene correlation hub in the lncRNA *AC002066.1* region, *CAV2*, has previously been shown to contribute to adipocyte hypertrophy in human cell line experiments.^73^ This motivated us to correlate adipocyte diameter measured in SAT biopsies from a Finnish BMI-discordant MZ twin cohort (see methods) with SAT expression of the 5 regional genes using SAT bulk RNA-seq data available in this cohort. In the correlation analyses, we found that the SAT bulk expression of the lncRNA, *AC002066.1*, was significantly positively correlated with the adipocyte diameter in a BMI-dependent way as a significant correlation was observed in the lower BMI twin siblings from each BMI-discordant MZ pair (adipocyte diameter: Spearman’s rho = 0.55, *p*_adj_ = 0.0023) (Figure 5E; Table S13), while the higher BMI twin siblings from each BMI-discordant MZ pair showed only nominally significant *p* values (Figure 5D; Table S13). Similarly, SAT bulk expression of *CAV2* also positively correlated with the adipocyte diameter in the lower BMI group (Spearman’s rho = 0.53, *p*_adj_ = 0.0043) (Figure 5F; Table S13). Overall, these results indicate that SAT expression of *AC002066.1* and *CAV2*, both unique adipocyte marker genes, is positively correlated with the adipocyte diameter, an indicator of adipocyte hypertrophy, in a BMI-dependent way given that our grouping of the BMI-discordant MZ twins controlled for the same genetic variants.

Taken together, we found putative evidence for selection with a north-south descending allele-frequency gradient of an abdominal obesity GWAS SNP, rs10494217, a predicted deleterious missense variant in a unique adipocyte marker gene, known adipose TF, and mouse thermogenesis gene, *TBX15*. The risk allele status of this *TBX15* missense variant also impacted the SAT adipocyte expression of 13 unique adipocyte marker genes consistently in two independent Finnish bariatric surgery cohorts, KOBS and RYSA. The adipocyte expression of one of the 13 genes, lncRNA *AC002066.1*, is significantly positively correlated with adipocyte expression of its several nearby genes, including a known adipocyte size-impacting gene, *CAV2*. Furthermore, SAT bulk expression of the lncRNA *AC002066.1* and *CAV2* were also positively correlated with adipocyte size measurements in a BMI-dependent way. These lncRNA results demonstrate putative downstream effects of the risk allele status of the abdominal obesity-associated GWAS missense variant, rs10494217, on adipocyte hypertrophy, a central cellular phenotype involved in obesity and its cardiometabolic consequences.

## Discussion

Variant-specific, cell-type expression changes contributing to abdominal obesity are not well understood yet. Although variants are known to affect SAT bulk tissue expression of genes as *cis-*expression quantitative trait loci (*cis-*eQTLs), the regulatory effects of abdominal obesity GWAS variants on gene expression have not been comprehensively assessed at the cell-type resolution. In the present study, we focus our analysis on an abdominal obesity GWAS missense variant, rs10494217, in a unique adipocyte marker and a known mouse thermogenesis gene, *TBX15*,^14^^,^^12^^,^^13^ to explore its role in adipocyte expression in the context of abdominal obesity. Using genotype data from the Finnish METSIM cohort, we found signals of selection for the derived T risk allele of rs10494217, which highlights an interesting case of selection acting on the abdominal obesity risk allele risen to a high frequency in the genetically isolated population of Finland.^17^^,^^63^ This may represent an example of an allele that likely provides beneficial adaptation to the cold, possibly due to its thermogenic effects in the cold, northern climate, but becomes metabolically harmful present-day when plenty of high-calorie food is available. We examined *TBX15* at the single-cell resolution by using SAT snRNA-seq data from two independent Finnish cohorts, and thus, our study largely circumvents the confounding factors related to cell-type heterogeneity in previous SAT bulk tissue analyses.^14^ We found that in SAT only adipocytes are enriched for abdominal obesity heritability and show a high level of expression for *TBX15*. We also identified 13 unique adipocyte marker genes, the adipocyte expression of which is impacted by the risk allele status of the missense variant in *trans,* consistently across two independent Finnish cohorts, thus exemplifying the gene expression changes induced by rs10494217 and the importance of *TBX15* for gene expression in adipocytes. Among the 13 *trans* genes, we found a lncRNA, *AC002066.1*, the adipocyte expression of which is correlated with the expression of its nearby genes, including *CAV2,* which has previously been implicated in adipocyte hypertrophy.^73^ We also found that SAT expression of both the lncRNA *AC002066.1* and *CAV2* are associated with adipocyte size in a BMI-dependent manner. Overall, we identified signals of selection acting on the derived allele of the abdominal obesity GWAS missense variant in *TBX15*, a master regulator of SAT gene expression, with downstream *trans* effects on adipocyte expression of multiple genes, including genes that link to adipocyte hypertrophy, a metabolically unhealthy consequence of obesity.

Obesity is highly polygenic,^74^ and there are well-documented population-based global differences in the prevalence of obesity, so it is crucial to examine prevalence of abdominal obesity GWAS risk alleles in different populations.^75^ We used genotype data from the Finnish METSIM cohort along with genotype data from tens of population groups in UKB to compare minor allele frequencies of the abdominal obesity GWAS variant rs10494217 across populations. We identified a global allele frequency spectrum of the T allele of rs10494217 as well as significant F_ST_ values based on empirical distributions between northern European, including the Finns, and other populations in UKB latitudinally. In addition, we performed a PGLS analysis, in which we found significant support for a model predicting allele frequency of rs10494217 with latitude, consistent with the F_ST_ results. Another example of selection on a latitudinal cline has previously been obtained with *TRPM8*, a gene implicated in tolerance to the cold and associated with migraine.^58^ This study exemplifies an allele that is selected for based on the environment, i.e., colder environment, while increasing the occurrence of migraine.^58^ Altogether, we provide converging evidence for a latitude-specific allele frequency spectrum of the rs10494217 abdominal obesity risk allele with the highest frequency in Finland.

We next observed a significant difference in LD surrounding rs10494217 between the Finns and Brits. This LD difference could be attributed to population structure or potentially allude to selection. To investigate this difference, we studied and identified signatures of selection of rs10494217 in Finns compared to other SNPs on chromosome 1 using EHH and iHS. As we were interested in examining how the haplotype breakdown for the derived and ancestral alleles of rs10494217 differs and how far the haplotype homozygosity extends, we first calculated the EHH and then iHS of rs10494217 and other SNPs, due to the higher power to detect selection provided by iHS when compared to EHH.^62^ The observed significant selection of the derived risk allele for abdominal obesity is an interesting example of selection acting on an SNP that is no longer beneficial and can explain the high MAF seen in Finns. The missense variant with the signal of selection resides within *TBX15*. We also performed a similar iHS analysis in UKB individuals from Sweden, Norway, Denmark, the UK, and Italy, which remained non-significant though a north-to-south trend was observed. Thus, among the tested populations in these iHS analyses, Finland was the only population with a significant selective signature for rs10494217.

In animal studies, *Ucp1* expression is significantly decreased when *Tbx15* is knocked out (KO) in the inguinal adipose depot in mice.^12^ Tbx15 has also been shown to play a role in the differentiation of brown and “brite” adipocytes in an *in vitro* murine study.^13^ It has previously been shown that Tbx15 plays a role in thermogenesis through the β-adrenergic pathway^12^ in the inguinal depot in mice, where beige adipocytes are known to reside.^76^ Thus, the global allele frequency and F_ST_ spectrum we observed may be attributed to higher prevalence of the T allele in colder climates, where it could potentially be beneficial for thermogenesis. Noteworthy, a previous study examining signals of selection in Greenlandic Inuit identified the *TBX15*/*WARS2* locus as having some of the highest genome-wide signatures of selection^77^ and concluded that selection at the *TBX15* locus in Inuit may be associated with adaption to the cold.^77^ The *TBX15* missense variant rs10494217 is not in LD (R^2^ = 0, using HaploReg v4.2, and R^2^ = 0.03 in the Finns of the METSIM cohort) with the variant rs4659153 that showed the highest iHS value in the *TBX15*/*WARS2* locus in Greenlandic Inuits.^77^ However, in human genetics, it is generally viewed as stronger evidence of an involvement of a gene in a monogenic disease when different populations have different mutations in the same gene accounting for the same phenotype. The same logic could be applied here, i.e., two populations with different population histories but similar climates may show separate signals for selection within *TBX15*, further signaling the significance of this gene in adaptation to the cold. Overall, the positive selection of the variant in *TBX15* in Finns could potentially relate to this known link between *Tbx15* and thermogenesis in mice^12^^,^^13^; however, future studies are warranted to thoroughly determine the role of TBX15 in thermogenesis in human fat cells.

As *TBX15* encodes for a TF, we examined effects of the *TBX15* missense variant on adipocyte gene expression in *trans*. Protein prediction tools identify rs10494217 to have a potentially damaging effect on the structure of TBX15, which in turn could affect binding of TBX15 to its target genes. Subsequently, we used SAT snRNA-seq data of two bariatric surgery cohorts from individuals with high BMI to identify 13 unique adipocyte marker genes impacted by the status of the risk allele T of the missense abdominal obesity GWAS variant in *trans*. One of the 13 *trans* genes, *BCL6*, is known to maintain thermogenic fitness in dormant brown adipocytes and oppose white adipocyte cellular identity.^78^ Another one of the 13 *trans* genes, *KLB*, has been found to have reduced expression levels in obesity, diabetes, and lipodystrophy,^79^ and impaired browning of white adipose tissue has also been observed with decreased gene dosage of *Klb* in cold-exposed mice.^79^ Thus, the function of these two genes aligns with the potential role of TBX15 in thermogenesis.^12^^,^^13^ As these experiments were done in mice, validation in human cell lines is warranted next to determine thermogenic effects of TBX15, BCL6, and KLB. Nevertheless, these previous results show how TBX15 can modify the thermogenic properties of adipocytes by affecting transcription of genes crucial for brown and beige adipocytes. Overall, the 13 genes exemplify gene expression changes induced by rs10494217 on multiple downstream genes in *trans* as well as provide potential TBX15 targets.

We observed that expression of one of the *trans* lncRNAs, *AC002066.1*, is correlated with expression of nearby genes using adipocyte pseudobulk data from the RYSA cohort. As lncRNAs have previously been shown to affect expression of nearby genes,^72^ *AC000266.1* may act on its nearby gene *CAV2*, a unique adipocyte marker gene. The high gene-gene correlation between *CAV2* and *AC002066.1* gene expression in adipocytes suggests a regulatory mechanism where *AC002066.1* may affect chromatin state or facilitate transcription of *CAV2*. Although *CAV2* showed only nominal significance by the rs10494217 risk allele status, this may be due to the individuals’ high BMI in both SAT snRNA-seq cohorts, since previous studies suggest that obesity affects adipose expression of *CAV2*.^80^ In our previous study,^14^ knocking down *TBX15* in human primary preadipocytes, we found that *CAV2* was significantly differentially expressed (DE) between the *TBX15* knocked down and control cells, showing a direct effect of TBX15 on *CAV2*. Cav2 has been shown to regulate adipocyte hypertrophy *in vitro*,^73^ and in line with this cellular study, we observed correlations between *AC002066.1* and *CAV2* SAT expression and adipocyte size that were stronger in the lower BMI group of the Finnish BMI-discordant MZ twin pairs. Taken together, we highlight a potential connection between a *TBX15* missense variant and adipocyte hypertrophy through *CAV2*, facilitated by the lncRNA *AC002066.1*.

One of the limitations of this study is the lack of ethnic diversity in UKB, resulting in relatively small sample sizes for some of the tens of populations in UKB. Another limitation is that we used obese Finnish cohorts for the analyses of the downstream effects of the *TBX15* missense variant on gene expression and thus, these results cannot directly be generalized to diverse populations or all BMI ranges. Considering samples sizes, despite the modest sample size of the KOBS snRNA-seq cohort, the 13 genes we identified should be robust as they were replicated in the independent RYSA cohort. We also note the limitation of the iHS statistic with a decreased power to detect selection at lower allele frequencies and the power estimated to be up to 10% with allele frequencies of 0.2–0.3.^62^ However, we did detect selection using this approach, as we found iHS values of greater than 2 for rs10494217, and iHS has been shown to outperform other statistics quantifying selective signatures, such as Fay and Wu’s H and Tajima’s D, in terms of power to detect selection.^62^ A further limitation of iHS is that it is testing for selective sweeps surrounding 30,000 years ago^81^ and thus, more recent selective sweeps will not be picked up on. Additionally, we did not perform a cross populational analysis of selection using the XP-EHH statistic because while both iHS and cross population EHH (XP-EHH)^82^ tests detect alleles that have risen to high frequency rapidly enough to show long-range association with nearby polymorphisms, the iHS test detects partial selective sweeps, whereas XP-EHH detects selected alleles risen to near fixation in one but not all populations.^82^ Furthermore, the abdominal obesity GWAS results may not be directly transferrable to other cohorts and populations due to differences in effect sizes, causal SNPs, and LD structure.^83^ Finally, the abdominal obesity GWAS we used comprises individuals of European descent without considering socioeconomic factors that are also known to affect obesity.^75^ Thus, there is a need for future, more diverse abdominal obesity GWASs that also assess socioeconomic factors.

In summary, we found evidence of selection in Finns for the risk allele T of an abdominal obesity GWAS SNP rs10494217, a missense variant in *TBX15*. Previous studies have found signatures of selection at the *TBX15* locus in Greenlandic Inuit, which they attributed to the potential role of TBX15 in thermogenesis.^77^ Our result builds on this finding by providing evidence of selection of another *TBX15* variant in the Finnish population, also living in a cold climate. Additionally, we analyzed *TBX15* at a single-cell resolution, focusing on the adipocyte cell type, as we observed that it is the relevant SAT cell type for the heritability of abdominal obesity in obese cohorts. We found replicated changes in adipocyte expression of 13 genes associated with the risk allele status of rs10494217, exemplifying how this potentially deleterious mutation could affect binding of *Tbx15* to its downstream targets in *trans*. We also discovered that adipocyte expression of one of the 13 *trans* genes, lncRNA *AC002066.1*, is correlated with a nearby gene, *CAV2*. Both *CAV2* and *AC002066.1* were in turn significantly correlated with adipocyte size, which links these two genes and adipocyte hypertrophy, a metabolically unhealthy consequence of obesity, in a human cohort. Finally, these results provide a framework for examining downstream effects of coding TF variants on single-cell expression as a mechanistic approach to identify *trans* genes instead of a standard *trans*-eQTL analysis that have remained poorly powered in human cardiometabolic tissues.^64^

## Data and code availability

The RYSA SAT snRNA-seq data, including the rs10494217 risk allele status, are available in NIH GEO, under accession number GSE274778. The iHS results for all SNPs genome-wide with a MAF > 0.05 using METSIM are available from the UCLA dataverse: https://doi.org/10.25346/S6/EVWWLG. No custom code was used, and all codes used for analyses in this study were unaltered from their publicly available sources, as outlined in the methods.

## Acknowledgments

We thank the participants of the KOBS, RYSA, CRYO and Twin cohorts, and the UK Biobank. This research was conducted using the UK Biobank Resource under application number 33934.

This study was supported by 10.13039/100000002NIH grants R01HG010505 (P.P.), R01HL170604 (P.P.), and R01DK132775 (P.P.); the 10.13039/501100002341Academy of Finland (272376, 266286, 314383, 335443 to K.H.P.; 314457 to A.J.; 338417 to S.H.); 10.13039/100008723Finnish Medical Foundation (K.H.P., A.J.); 10.13039/501100013500Finnish Diabetes Research Foundation (S.H., K.H.P.); Orion Foundation (S.H.); 10.13039/501100009708Novo Nordisk Foundation (NNF10OC1013354, NNF17OC0027232, NNF20OC0060547 to K.H.P.); 10.13039/501100007417Paulo Foundation (S.H., K.H.P.); Gyllenberg Foundation (K.H.P.); 10.13039/501100006306Sigrid Jusélius Foundation (K.H.P.); 10.13039/501100008484Paavo Nurmi Foundation (S.H.); Helsinki University Hospital Research Funds (S.H., K.H.P., A.J.); and the 10.13039/100007797University of Helsinki (K.H.P.).

## Author contributions

M.D., A.K., S.H.T.L., and M.A. performed the computational analysis of the data. S.H., D.K., V.M., B.W.v.d.K., U.S., T.S., A.J., J.P., M.L., and K.H.P. recruited the Finnish cohorts and/or collected the samples. S.H.T.L., M.A., S.R., S.H., U.T.A., M.U.K., and P.P. generated the multi-omics data. M.D., A.K., and P.P. conceptualized the study, and P.P. supervised the work. M.D., A.K., and P.P. wrote the manuscript. All authors read, reviewed, and/or edited the manuscript. All authors read and approved the final manuscript.

## Declaration of interests

The authors declare no competing interests.

## Web resources

GenBank, https://www.ncbi.nlm.nih.gov/genbank/
GEO, https://www.ncbi.nlm.nih.gov/geo/

## Supplemental information

Data S1. Tables S1–S13

Document S2. Article plus supplemental information
