# Supplementary material for: An abdominal obesity missense variant in the adipocyte thermogenesis gene TBX15 is implicated in adaptation to cold in Finns
Source: Am J Hum Genet. 2024 Nov 7;111(11):2542–60. doi: 10.1016/j.ajhg.2024.10.001 (PMC11568758; doi:10.1016/j.ajhg.2024.10.001)
Supplement: Document S1. Figures S1 and S2 [file mmc1.pdf]

The American Journal of Human Genetics, Volume *111*

Supplemental information

An abdominal obesity missense variant in the adipocyte thermogenesis gene *TBX15* is implicated in adaptation to cold in Finns

Milena Deal, Asha Kar, Seung Hyuk T. Lee, Marcus Alvarez, Sandhya Rajkumar, Uma Thanigai Arasu, Dorota Kaminska, Ville Männistö, Sini Heinonen, Birgitta W. van der Kolk, Ulla Säiläkivi, Tuure Saarinen, Anne Juuti, Jussi Pihlajamäki, Minna U. Kaikkonen, Markku Laakso, Kirsi H. Pietiläinen, and Päivi Pajukanta

Supplemental Figures

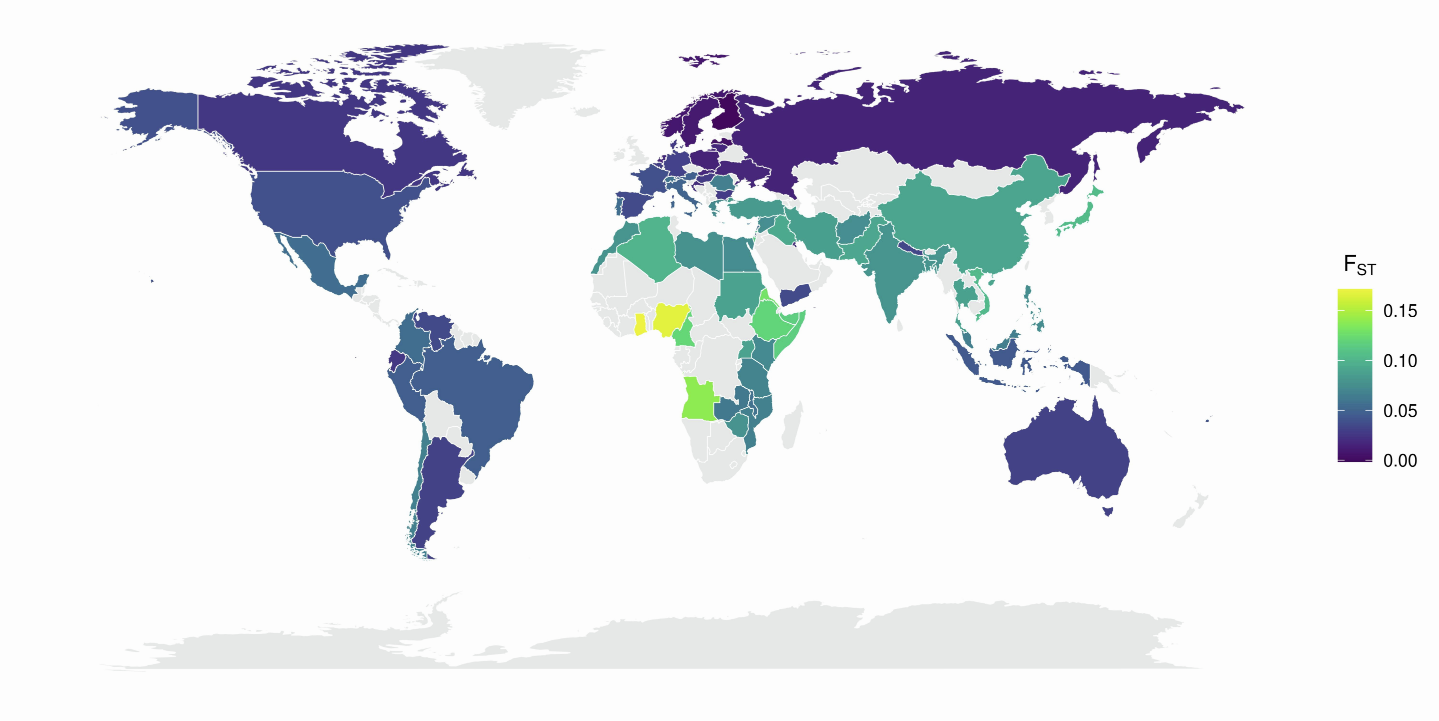

Figure S1. Increased F_{ST} values indicate differences in allele frequency between Finns and populations closer to the equator. F_{ST}, fixation index, of rs10494217 between the Finns in METSIM (n=6,738) and the populations in UKB with *n*>30.

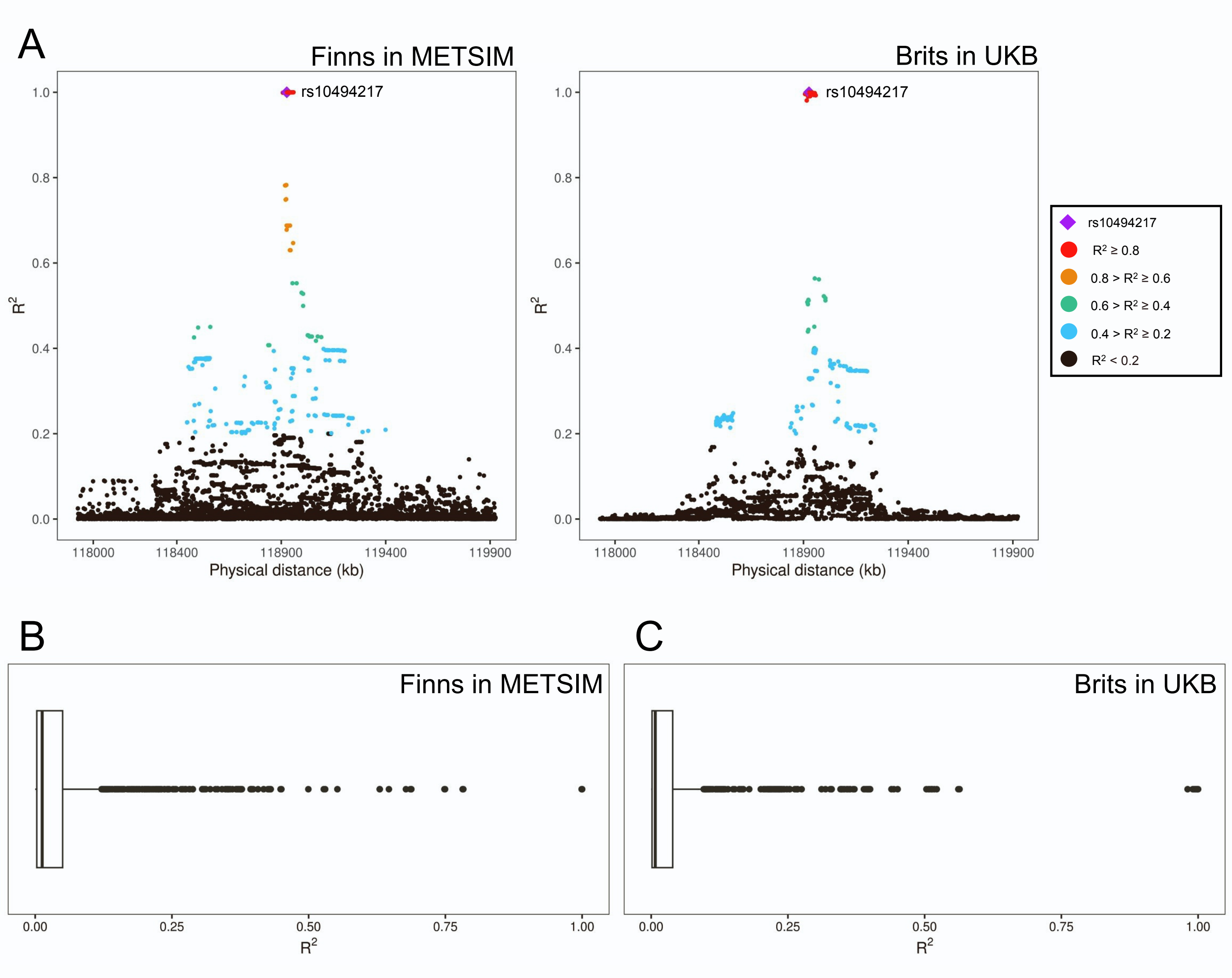

Figure S2. Linkage disequilibrium (LD) between rs10494217 and nearby SNPs differs between the Finns and Brits. LD was calculated for all SNPs residing within 1 Mb on each side of rs10494217. LD was calculated using the unrelated Finns from METSIM (*n*=6,738) and Brits from the UK Biobank (UKB) (*n*=6,738). A) LD is shown for 1 Mb on each side of rs10494217 for the Finns from METSIM and Brits from UKB. Boxplot of the LD between rs10494217 and the SNPs within 1 Mb of rs10494217 on either side for B) the Finns from METSIM and C) the Brits from UKB. The *P* for the difference in LD of rs10494217 and the SNPs extending 1 Mb on each side of rs10494217 between the Finns and Brits is 1.31×10^{-41}, with the stronger LD observed in the Finns.
